# General Perceptions and Awareness Level among Local Residents in Penang Island toward Bats Conservation Efforts

**DOI:** 10.21315/tlsr2017.28.2.3

**Published:** 2017-07-31

**Authors:** Nur Juliani Shafie, Shahrul Anuar Mohd Sah, Aini Hasanah Abdul Mutalib, Nik Fadzly

**Affiliations:** 1 School of Biological Sciences, Universiti Sains Malaysia, 11800 USM Pulau Pinang, Malaysia; 2 Centre for Marine & Coastal Studies (CEMACS), Universiti Sains Malaysia, 11800 USM Pulau Pinang, Malaysia

**Keywords:** Chiroptera, Community-based Conservation Strategy, Demography, Penang

## Abstract

The population of bats has declined from year to year caused by human activities such as logging and hunting activities. Since the human factor is linked to the issues of population decline in many animal species, a community-based conservation strategy that involved local communities is needed. We conducted face-to-face surveys among residents in Penang Island to assess knowledge and awareness level toward bats conservation efforts. We collected demographic values such as age, gender, level of education, length of residency as well as their monthly income, since different group in these variable might have different perception. We found that age groups, level of education and monthly income have shown significant differences among the respondents. However, no other significant differences were indicated for by gender and length of residency. Respondent’s knowledge of bats showed that the majority of the respondents were less likely to value the importance of bats in the ecosystem. We recommended stronger legal system, earlier exposure towards environmental education, well-planned urbanisation implementation and long-term monitoring programs to strengthen efforts in conserving bats in Malaysia.

## Abstract (ru)

Populasi kelawar telah berkurangan dari tahun ke tahun disebabkan oleh aktiviti manusia seperti aktiviti pembalakan dan pemburuan. Oleh kerana faktor manusia dikaitkan dengan isu penurunan populasi dalam banyak spesies haiwan, strategi pemuliharaan berasaskan komuniti yang melibatkan masyarakat setempat diperlukan. Satu tinjauan secara bersemuka telah dibuat dalam kalangan penduduk di Pulau Pinang untuk menilai tahap pengetahuan dan kesedaran terhadap usaha pemuliharaan kelawar. Nilai-nilai demografi seperti umur, jantina, tahap pendidikan, tempoh masa pemastautin serta pendapatan bulanan mereka dikumpul, kerana kumpulan yang berbeza dalam pemboleh ubah ini mungkin mempunyai persepsi yang berbeza. Kajian mendapati bahawa kumpulan umur, tahap pendidikan dan pendapatan bulanan menunjukkan perbezaan yang ketara di kalangan responden. Walau bagaimanapun, tiada perbezaan penting lain yang ditunjukkan oleh jantina dan tempoh masa pemastautin. Pengetahuan tentang responden kelawar menunjukkan bahawa majoriti responden kurang mengutamakan pentingnya kelawar dalam ekosistem. Kajain mengesyorkan sistem perundangan yang lebih kuat, pendedahan terdahulu terhadap pendidikan alam sekitar, pelaksanaan perbandaran yang dirancang dengan baik dan program pemantauan jangka panjang untuk menguatkan usaha memelihara kelawar di Malaysia.

## INTRODUCTION

The habitat loss and fragmentation of forest by human are recognised as important factors in influencing the decline of forest-dependent fauna (Kingston 2010). Southeast Asia, known as one of the world’s biodiversity hotspots of bats faces the same issue. Besides habitat loss, unregulated hunting of bats is also reported as primary reasons for the declining of bat abundance (Mohd-Azlan *et al*. 2001). Bats, which considered as wild exotic meat, are widely consumed in urban areas, especially in Southeast Asia (Bennett & Rao 2002). In Thailand, the world’s smallest bat species, *Craseonycteris thonglongyai* were dried and sold as souvenirs to the foreigners (Robinson 1995). Similarly, mounted specimens of many bat species were sold in souvenir shops in Vietnam (Lee *et al.* 2015) and bats were also trades in several markets in Laos (Francis *et al*. 1999).

In Malaysia, the problems are similar. According to IUCN (2012), the population of Malaysian bats are decreasing in 26% of species and only 15% are still stable. In Sarawak, bats are shot by the Iban for sport or to eradicate bats from fruit plantations (Fujita & Tuttle 1991). Besides hunting, the primary threats to bats species include habitat loss and degradation through logging (Kingston *et al*. 2012). As human activities cause more destructive impact than natural effect, conservation efforts and a community-based conservation effort must be increased (Abd Mutalib *et al*. 2013).

However, creating a conservation education to connect between people with nature is not easy (Reynolds & Braithwaite 2001; Abd Mutalib *et al*. 2013). Finding a balance between monetary with conservation value might be difficult, and requires an in-depth research especially on the areas’ of carrying capacity, demographic structures, and conservation interests (Humavindu & Stage 2014). Social demographics such as age, gender, level of education, monthly income and years at residence play an important role in the determination of the level of awareness towards wildlife and often act as behavioural predictors (Thornton & Quinn 2009; Loyd & Miller 2010; Mahmood-ul-Hassan *et al*. 2011; Shumway *et al*. 2014).

This study was conducted in Penang Island, Malaysia that covers an area of 285 km^2^ (Pradhan *et al*. 2012). This island is known as tourist hotspot and the second most important urban centre in Malaysia (Teo 2003). Unfortunately, the habitat loss due to urbanisation in Penang Island is rapidly increasing from year to year. Bats are responsive to urbanisation and are useful bioindicators of habitat quality (Fenton 2003). They have slow life histories (Barclay & Harder 2003) and are slow to recover from habitat change (Racey & Entwistle 2003). Since bats are specialist, they depend on the presence of trees for foraging and roosting in order for them to survive (Mayle 1990). Thus, conservation effort is urgently needed to reduce the impact of these threats on the bat species in Penang Island.

However, conservation efforts for the non-game and non-charismatic wild creatures such as bats should not only focus on the environmental issues, but also on social and cultural matters (Enriquez & Mikkola 1997). Assessing resident’s perception and level of awareness toward bats in general based on their demographic structures, can provide important insight into how to improving urban wildlife management. Furthermore, the general public attitude towards bats has not been investigated extensively throughout the world (Mahmood-ul-Hassan *et al*. 2011). Thus, the purpose of this study are: (1) to determine the general perceptions and the level of awareness among local residents in Penang Island based on their age groups, gender, level of education, years at residence and monthly income and (2) to determine the conservation actions that local residents would be willing to take for future conservation program.

## MATERIALS AND METHODS

**Figure 1:**
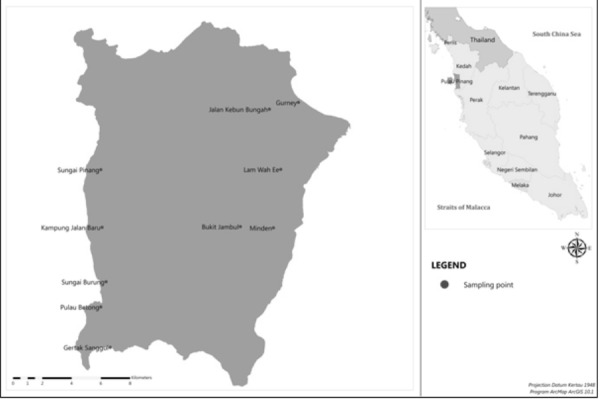
Map of the study sites where surveys were carried out in Penang Island, Malaysia.

We conducted a social survey in a total of ten sampling sites in Penang Island; Persiaran Gurney (N 5°25′59.88″, E 100°18′55.80″), Jalan Kebun Bungah (N 5°25′44.40″, E 100°17′51.42″) Jalan Lam Wah Ee (N 5°23′30.66″, E 100°18′17.22″), Minden (N 5°21′20.52″, E 100°18′1.80″), Bukit Jambul (N 5°21′22.85″, E 100°16′48.34″), Kampung Sungai Pinang (N 5°23′28.02″, E 100°11′38.34″), Kampung Jalan Baru (N 5°21′19.77″, E 100°11′41.50″), Kampung Sungai Burung (N 5°19′18.71″, E 100°11′51.09″), Pulau Betong (N 5°18′22.68″, E 100°11′39.84″) and Gertak Sanggul (N 5°16′52.93″, E 100°12′0.79″) (Fig. 1). Persiaran Gurney, Jalan Kebun Bungah, Jalan Lam Wah Ee, Minden and Bukit Jambul were located in urban area, while Kampung Sungai Pinang, Kampung Jalan Baru, Kampung Sungai Burung, Pulau Betong and Gertak Sanggul were located in rural area.

We have developed a semi structured set of questionnaires for 150 respondents in Penang Island in 2014. We conducted the survey in Malay language, since most of the houses in the sites were predominantly by Malay native speakers. Enumerators that we have selected to assist the survey are highly understood on the concerns of bats’ conservation and able to explain it to the respondents if necessary. Since the enumerators have hands-on experience in bats study, the enumerators were required to simply explained to the respondents on the impact of bats’ conservation and the importance of this study. Respondents were chosen opportunisticaly and volunteer-based. Refusal to answer the questionnaire was usually due to insufficient of time. We chose our respondents based on several criteria. Only one respondent was chosen per home, respondents must be 18 years old and above, and agree to answer the questionnaires. We chose to use face-to-face surveys to allow the better understanding among the residents, with casual and guided manner (Doyle 2005; Senko *et al*. 2011). The questionnaire was designed to take less than ten minutes to complete and comprised both multiple-choice and short answer questions (Shumway *et al*. 2014). The data collection was confidential and the personal data of the respondents were not recorded (Abd Mutalib *et al*. 2013; Senko *et al*. 2011).

Our aims were to determine the general perceptions and level of awareness based on their demographic structure and also their willingness to join in future conservation program. Demographic structure of this study comprise several variables such as age, gender, level of education, length of residency as well as their monthly income. We took into account these variables since different parameters within the variables might have different perception on local conservation (Dietz *et al*. 1998; Zelezny *et al*. 2000). Questionnaires comprised two sections, which are section (A) and section (B). Section (A) revolved around the demographic structure of respondents, while section (B) focused on the knowledge, perception and willingness to be involved in bats’ conservation efforts.

Evaluation was done from each questionnaire answered by respondents using a specific scoring system based on social research methods by Bernard (2012) (Appendix 1). Respondents were given boxes to tick for multiple choice questions. We also inquired respondents to elaborate their choices for some questions. For questions that required respondent to tick as many as apply, marks were given for each box implicating the exposure that the respondents have received on bats conservation issues. Scoring system can be summarised as:

$$\frac{\text{Marks of respondents}}{90\;(\text{full marks of the whole questions})}\;(*)\;100$$

All data were tested for normality. Since the data is normally distributed (*p* > 0.05), we used parametric data such as one-way Analysis of Variance (ANOVA) to determine the data groups that varied significantly and Post-hoc Tukey test was then applied for each significant variable. All statistical analyses were performed by using a Microsoft Excel 2010 and JMP Pro 10.

## RESULTS

**Figure 2:**
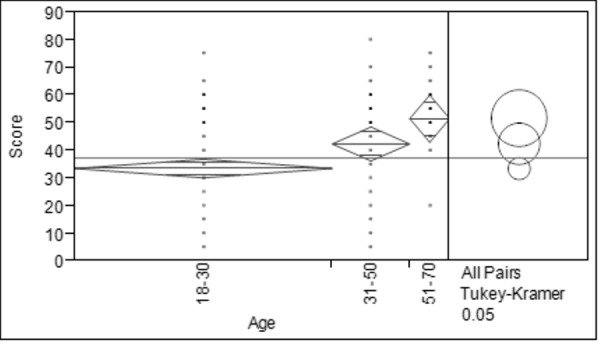
Mean marks of the respondents based on age groups.

**Figure 3:**
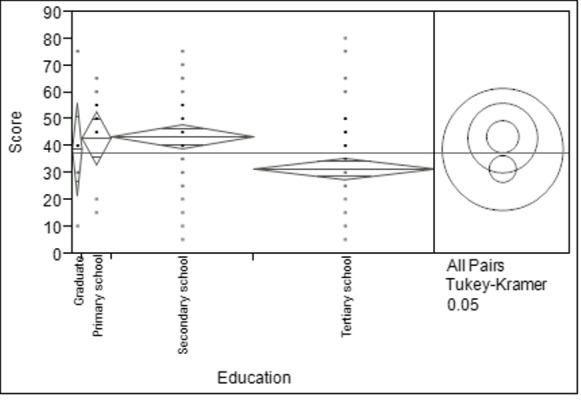
Mean marks of the respondents based on education level.

**Figure 4:**
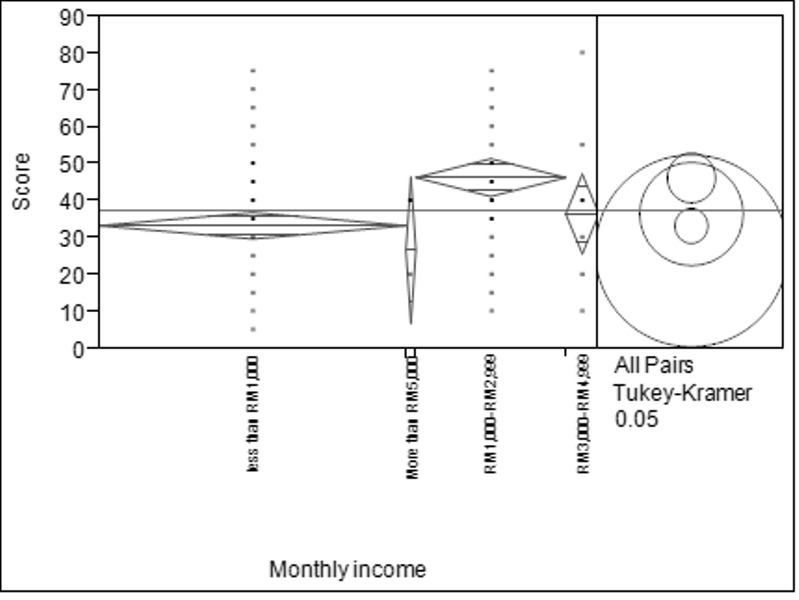
Mean marks of the respondents based on monthly income.

**Figure 5:**
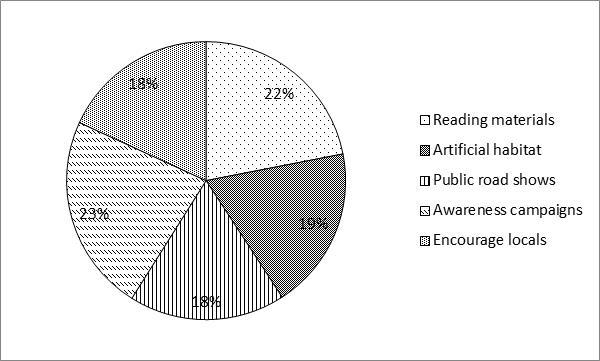
Respondents willingness to participate in possible conservation actions to mitigate threats.

There was a significant difference between age groups among respondents as determined by one-way ANOVA (F_(2, 150)_ = 9.272, *p* < 0.05). Respondents aged between 51–70 years old obtained the highest score, while respondents aged between 18–30 years old showed the lowest score (Fig. 2). Figure 3 shows that there was also a statistical significance for level of education among respondents as determined by one-way ANOVA (F_(3, 150_) = 5.727, *p* < 0.05). Results revealed that respondents with level of secondary school was significantly higher (mean = 43.475 ± 2.263) and respondents with level of tertiary school was significantly lower (mean = 31.467 ± 2.007). Based on Figure 4, there was a statistically significant difference between respondent’s monthly income as determined by one-way ANOVA (F_(3,150)_ = 6.087, *p* < 0.05). Tukey post hoc test revealed that respondents with monthly income between RM1,000 to RM2,999 was significantly different (mean = 46.333 ± 2.582) compared to respondents with monthly income of less than RM1,000 (mean = 33.315 ± 1.806). When respondents were asked about their opinion and their willingness to participate in conservation actions for bats, 23% of the respondents willing to join in awareness campaigns, 22% in distributing reading materials (e.g. brochures, magazines, newspaper and articles) on conservation of bats, followed by building artificial habitat for wild bats (19%) and both public road shows and encourage local with 18% respectively (Fig. 5).

## DISCUSSION

Demographic structure is known as an important factor to be considered to increase the level of awareness and perceptions among local communities (Abd Mutalib *et al*. 2013). One of our key findings was that respondents aged at 51–70 years old have the highest score compared to respondents aged at 18–30 years old. A majority of the respondents aged at 51–70 years old were unemployed or pensioners with monthly income between less than RM1,000 to RM 2,999 and they lived in rural area (62.5%). When asked, respondents aged at 51–70 years old that were unemployed obtained their monthly income given by their children. The oldest age group seemed to be mostly aware of the bat conservation effort in accordance with other study describing that level of awareness of current wildlife and conservation issues increased as age increased (Thornton & Quinn 2009). In addition, older respondents lived in rural areas have more exposure to wildlife and they valued nature more than people lived in urban area (Thornton & Quinn 2009; Shumway *et al*. 2014). The respondents aged between 18–30 years have the lowest score and this result is similar to previous study that suggested younger respondents lack of desire of to learn about wildlife (Thornton & Quinn 2009). The majority of them live in urban areas (62.1%) since accessibility is much wider, especially for career-wise. When asked, younger respondents mostly have given time constraint as excuse that limited their opportunity to be concerned and observant towards environmental and wildlife issues. According to Miller and Hobbs (2002), individuals might assess and value wildlife depending on their daily routine, especially where they live and work.

In terms of education level, respondents with secondary school level and aged between 18–30 years old (N = 30, 20.0%) obtained the higher score. The majority of the respondents with secondary school level and aged between 18–30 years old were in science stream (80%). In science stream for secondary school, respondents were taught about general science and biology specifically about knowledge of organisms. This results parallel to work done by Tikka *et al.* (2000) that found students of biology exhibited the most positive attitudes and the greatest level of knowledge concerning the environment. They also participated in many nature-related activities compared to students in other educational backgrounds. Study by Yilmaz *et al*. (2004) that focused on the views of elementary and middle school Turkish students toward environmental issues indicated that students with high exposure in science courses resulted in more positive attitudes toward environmental issues. Previous studies also proposed that environmental knowledge is an essential precursor of attitude formation (Kellert & Westervelt 1984; Kaiser *et al*. 1999) and increasing environmental knowledge may result in more positive pro-environmental attitudes. Thus, the higher level of education would not necessarily reflect the positive attitudes towards environment and wildlife issues, but the environmental knowledge is more important in shaping their positive attitudes towards environment.

Another parameter, the monthly income has shown the significant difference in showing the respondent’s score of awareness regarding bat conservation. We found that there was a relationship between the monthly income and the level of education in this study. Most of the respondents with monthly income less than RM1,000 had at least secondary school level. Meanwhile, respondents with monthly income between RM1,000 to RM2,999 had at least tertiary school level. However, the result in this study showed that higher monthly income would not necessarily lead to increase of level of awareness among local communities.

Based on behavioural theory, the human interest to change behaviour was depending on their positive perception and attitude toward wildlife (Vaske & Donnelly 2007). Besides, human attitudes toward most of the animals are influenced particularly by the species of animal (Mahmood-ul-Hassan *et al*. 2011). People throughout the world love colourful birds and pet animals such as cats and dogs, but they dislike invertebrates such as bats, rats, reptiles and owls (Bjerke & Østdahl 2004). The negative perception and attitude toward bats probably due to the fact that bats are small and unfamiliar to humans both behaviourally and morphologically (Davey 1994), bats are considered as pests, symbol of bad omen and are also considered as useless creatures in most people’s view (Mahmoodul-Hassan *et al*. 2011).

There was no significant difference between the score from the groups of male and female respondents as we developed this social survey. While Dietz *et al*. (1998) and Zelezny *et al*. (2000) indicated that female have more willingness to be involved in the nature conservation in general, we indicated that the similarities between the female and male respondents were based on few reasons. Since women in urban areas area more independent and mostly are working, obligation towards financial gain was almost similar to men. Thus, willingness to get involved might be similar to male respondents due to time constraints and lack opportunities. Unlike green sea turtles (*Chelonia mydas*) (Abd Mutalib *et al*. 2013) whose parts can be consumed such as meat and eggs, bats are not consumable and presumed as insignificant towards family environment and diet.

As recommendation, efforts in conserving bat species should be concentrated on improving the legal status of bats, and such legislation should reflect the special needs of bats species. The urbanisation in urban area such as in Penang Island also should be well-planned since habitat loss is the most urgent threats to bat survival. We suggested that in the future, younger respondents will be more exposed to practical environmental education (EE) instead of only been taught about theory in the class to increase their awareness for the endangered animals. The role of mass media such as newspapers, television and the use of social media such as Facebook, Twitter and Instagram could also spread the message of the importance of bats. Inventories and long-term monitoring are needed for important sites to determine species distribution, habitat requirements and also key areas for protection. Strong collaboration between researchers, government agencies, non-governmental organisation (NGO), and most importantly participation by local citizens are vital in order to achieve successful conservation programmes.
